# Pre-operative beta-blocker therapy does not affect short-term mortality after esophageal resection for cancer

**DOI:** 10.1186/s12893-020-01017-x

**Published:** 2020-12-22

**Authors:** Souheil Reda, Rebecka Ahl, Eva Szabo, Erik Stenberg, Maximilian Peter Forssten, Gabriel Sjolin, Yang Cao, Shahin Mohseni

**Affiliations:** 1 Division of Upper Gastrointestinal Surgery, Department of Surgery, Orebro University Hospital, Orebro, Sweden; 2 School of Medical Sciences, Orebro University, Orebro, Sweden; 3 Division of Trauma and Emergency Surgery, Department of Surgery, Karolinska University Hospital, Stockholm, Sweden; 4 Division of Surgery, CLINTEC, Karolinska Institutet, Stockholm, Sweden; 5 Division of Trauma and Emergency Surgery, Department of Surgery, Orebro University Hospital, Orebro, Sweden; 6 Clinical Epidemiology and Biostatistics, School of Medical Sciences, Orebro University, Orebro, Sweden

**Keywords:** Beta-blocker, Esophageal cancer, Beta-blocker in surgery, Mortality

## Abstract

**Background:**

It has been postulated that the hyperadrenergic state caused by surgical trauma is associated with worse outcomes and that β-blockade may improve overall outcome by downregulation of adrenergic activity. Esophageal resection is a surgical procedure with substantial risk for postoperative mortality. There is insufficient data to extrapolate the existing association between preoperative β-blockade and postoperative mortality to esophageal cancer surgery. This study assessed whether preoperative β-blocker therapy affects short-term postoperative mortality for patients undergoing esophageal cancer surgery.

**Methods:**

All patients with an esophageal cancer diagnosis that underwent surgical resection with curative intent from 2007 to 2017 were retrospectively identified from the Swedish National Register for Esophagus and Gastric Cancers (NREV). Patients were subdivided into β-blocker exposed and unexposed groups. Propensity score matching was carried out in a 1:1 ratio. The outcome of interest was 90-day postoperative mortality.

**Results:**

A total of 1466 patients met inclusion criteria, of whom 35% (n = 513) were on regular preoperative β-blocker therapy. Patients on β-blockers were significantly older, more comorbid and less fit for surgery based on their ASA score. After propensity score matching, 513 matched pairs were available for analysis. No difference in 90-day mortality was detected between β-blocker exposed and unexposed patients (6.0% vs. 6.6%, p = 0.798).

**Conclusion:**

Preoperative β-blocker therapy is not associated with better short-term survival in patients subjected to curative esophageal tumor resection.

## Background

Esophageal tumor resection is a high-risk surgical procedure with substantial risk of postoperative mortality [1]. Despite measures taken to improve outcomes, including centralization, multidisciplinary team approach to treatment, increasing laparoscopic and endoscopic procedures, and standardization of chemo- and radiotherapy protocols, esophageal cancer remains a diagnosis with a relatively poor prognosis [1]. Morbidity rates after esophageal cancer surgery remain up to 50% and early postoperative mortality rates up to 10% have been reported [2].

For decades it has been known that surgical insults lead to an increase in serum catecholamine levels with associated increases in adverse cardiovascular postoperative events, a rise in inflammatory responses, insulin resistance and unfavorable immunomodulatory changes [3, 4]. Several studies have shown significant benefits of β-blocker therapy in the context of non-cardiac surgery in which β-blocker treatment reduces adverse postoperative events with a significant decrease in postoperative mortality [5–8]. It has been postulated that better outcomes are due to a reduction in the spike in adrenergic activity, which offers a cardio-protective effect and a reduction in the inflammatory responses initiated by the surgical insult [8]. A special aspect of esophageal cancer surgery, compared to other cancer surgeries, is the use of postoperative epidural thoracic analgesia (TEA), which has independently been associated with a decrease in postoperative morbidity [9].

This nationwide cohort study aims to investigate the association between regular preoperative β-blocker therapy and 90-day all-cause mortality after esophageal resection surgery for cancer. The authors hypothesize that β-blocker exposure would be associated with a reduced risk of mortality following esophageal cancer resection.

## Methods

### Patient identification

Following ethical approval from the Regional Review Board (Ref. 2018–361, Uppsala County, Sweden), all patients diagnosed with esophageal cancer from 1 January 2007 to 31 December 2017 were identified from the Swedish National Register for Esophagus and Gastric Cancers (NREV). NREV is a prospectively collected, externally validated national registry for patients diagnosed with esophageal and gastric cancers in Sweden [10]. It contains surgical and oncological data for both curative and palliative cancer patients. The registry is synchronized with other national registries maintained by the National Board of Health and Welfare. This is possible because all Swedish residents have a unique personal identification number which is used by both healthcare providers and governmental agencies. This enables the NREV registry to capture co-morbidity data using the International Statistical Classification of Diseases (ICD-codes) and prescribed drugs with Anatomical Therapeutic Chemical Classifications (ATC) codes from both primary and secondary care providers. The registry also contains information on date of death based on data from the Swedish Population Registry covering all deaths among Swedish citizens and those with a Swedish personal identification number. The following variables were included in the data analysis: sex, age, American Society of Anesthesiologist (ASA) classification, Charlson Comorbidity Index (CCI), neoadjuvant oncological therapy, type of thoracic surgical approach, pTNM-classification, and postoperative mortality at 90 days.

In the current study, selection of patients was based on surgical ICD-codes (JCC00-JCC97) including curative cardia cancer class 1 and 2 according to Siewert-Stein classification [11]. Only squamous cell carcinoma, adenocarcinoma, or dysplastic tumors based on the preoperative and postoperative histopathology were included in the study. The outcome of interest was 90-day postoperative mortality.

### Beta-blocker therapy

ATC codes (specifically ATC C07) were used to extract information on beta-blocker prescriptions from the registry. Patients who received a beta-blocker prescription preadmission for their esophageal cancer resection surgery were coded “β-blocker exposed”. β-blocker agents were not sub-grouped according to dose or type. Information on drug indication was not collected as this variable is not listed by the drug registry.

An assumption was made that patients who preoperatively were on β-blocker therapy also continued to receive their beta-blocker agent both in-hospital and following discharge. This assumption was made on the basis of national guidelines set by the Swedish Society of Anesthesiology and Intensive Care regarding preoperative drug administration protocols in regard to ongoing preoperative β-blocker therapy. Such guidelines are also supported internationally by the guidelines set by the American College of Cardiology and American Heart Association (ACC/AHA) where discontinuation of a regular beta-blocking agent is actively discouraged [12, 13].

### Statistical analysis

Descriptive statistical methods were used for patients’ characteristics and outcomes. Patients receiving β-blocker therapy were matched in a 1:1 ratio to a control group of patients not receiving β-blocker therapy preoperatively, using propensity score matching. Pairs were matched using optimal matching of weights rather than nearest neighbor. Variables included in the propensity score match were sex, age, ASA classification, CCI, neoadjuvant oncological therapy, type of thoracic surgical approach, histopathology and pTNM classification. Before matching, a Chi-squared test and Fisher's exact test were used for categorical variables, while a Student's t-test was used for continuous variables to determine statistical significance between the cohorts. After matching, McNemar's test was used for categorical variables while a paired Student's t-test was used for continuous variables to determine statistical significance. In the cases where a variable had more than two categories, a 2 × 2 contingency table was created for each category within the variable with the remaining categories being combined into one group. McNemar's test was performed on each contingency table and the resulting p-values were averaged. Finally, the p-values were adjusted for multiple testing using Bonferroni correction. In order to validate the results of the propensity score matching, conditional Poisson regression analysis was performed adjusting for age, sex, ASA classification, CCI, neoadjuvant oncological therapy, type of thoracic surgical approach, histopathology, and pTNM classification. Results were considered statistically significant at a p value of less than 0.05. Analysis were carried out using Statistical Package for Social Sciences version 25 (IBM, Armonk, New York) and R statistical programming language (Table 1).

**Table 1:** Demographics for the total cohort

Variable	Total cohort (*n* = 1466)
*Patient characteristics*	
Age	
Mean (SD)	65.0 (9.46)
Median [Min, Max]	66.0 [0, 88.0]
Sex	
Male	1181 (80.6%)
Female	285 (19.4%)
ASA-classification	
ASA-1	521 (35.5%)
ASA-2	735 (50.1%)
ASA-3	207 (14.1%)
ASA-4	3 (0.2%)
CCI	
< = 4p	683 (46.6%)
5–6p	650 (44.3%)
> = 7p	133 (9.1%)
Type of histology	
Adenocarcinoma	1159 (79.1%)
Squamous cell carcinoma	307 (20.9%)
T-classification	
Tx	135 (9.2%)
T0	2 (0.1%)
T1	98 (6.7%)
T2	402 (27.4%)
T3	773 (52.7%)
T4	56 (3.8%)
N-classification	
N0	794 (54.2%)
N1	517 (35.3%)
N2	98 (6.7%)
N3	17 (1.2%)
Nx	40 (2.7%)
M-classification	
M0	1398 (95.4%)
M1	30 (2.0%)
Mx	38 (2.6%)
Thoracic surgical approach	
Right-sided thoracotomy	1129 (77.0%)
Thoracoscopy	221 (15.1%)
Other	116 (7.9%)
Neoadjuvant treatment	
Yes	952 (64.9%)
No	508 (34.7%)
Missing	6 (0.4%)

## Results

A total of 1446 patients met inclusion criteria during the specified study period. Thirty-five percent (n = 513) of the study cohort were receiving β-blocker therapy prior to their cancer resection surgery. The β-blocker exposed group was significantly older (67.8 [SD 8.2] vs. 63.5 [SD 9.8] years, p < 0.001) and classified as less fit for surgery based on a larger proportion of patients with ASA classification of 3 and 4 (23.4% vs. 9.4%, p < 0.001) (Table 2). There were no statistically significant differences in regard to the distribution of surgical procedures and TNM classification between the groups. Neoadjuvant oncological therapy was more common in the β-blocker negative cohort but did not reach statistical significance (66.7% vs. 61.6%, p = 0.05) (Table 2).

**Table 2:** Demographics and outcomes prior to and following propensity score matching

	Before matching			After matching		
	BB^(−)^	BB^(+)^	*p*	BB^(−)^	BB^(+)^	*p*
	(*n* = 953)	(*n* = 513)		(*n* = 513)	(*n* = 513)	
*Patient characteristics*						
Sex			0.12			0.613
Male	756 (79.3%)	425 (82.8%)		418 (81.5%)	425 (82.8%)	
Female	197 (20.7%)	88 (17.2%)		95 (18.5%)	88 (17.2%)	
Age in years, mean (SD)	63.5 (9.8)	67.8 (8.2)	< 0.001	66.9 (8.3)	67.8 (8.2)	0.085
CCI			< 0.001			0.468
< = 4p	525 (55.1%)	158 (30.8%)		182 (35.5%)	158 (30.8%)	
5–6p	379 (39.8%)	271 (52.8%)		284 (55.4%)	271 (52.8%)	
> 7p	49 (5.1%)	84 (16.4%)		47 (9.2%)	84 (16.4%)	
ASA-classification			< 0.001			1.00
ASA-1	432 (45.3%)	89 (17.3%)		89 (17.3%)	89 (17.3%)	
ASA-2	431 (45.2%)	304 (59.3%)		335 (65.3%)	304 (59.3%)	
ASA-3	89 (9.3%)	118 (23.0%)		88 (17.2%)	118 (23.0%)	
ASA-4	1 (0.1%)	2 (0.4%)		1 (0.1%)	2 (0.4%)	
Histological classification			0.032			0.506
Adenocarcinoma	737 (77.3%)	422 (82.3%)		413 (80.5%)	422 (82.3%)	
Squamous cell carcinoma	216 (22.7%)	91 (17.7%)		100 (19.5%)	91 (17.7%)	
T-classification			0.20			1.00
Tx	81 (8.5%)	54 (10.5%)		49 (9.6%)	54 (10.5%)	
T0	0 (0.0%)	2 (0.4%)		0 (0.0%)	2 (0.4%)	
T1	58 (6.1%)	40 (7.8%)		32 (6.2%)	40 (7.8%)	
T2	262 (27.5%)	140 (27.3%)		138 (26.9%)	140 (27.3%)	
T3	514 (53.9%)	259 (50.5%)		276 (53.8%)	259 (50.5%)	
T4	38 (4.0%)	18 (3.5%)		18 (3.5%)	18 (3.5%)	
N-classification			0.94			1.00
NX	25 (2.6%)	15 (2.9%)		15 (2.9%)	15 (2.9%)	
N0	524 (55.0%)	270 (52.6%)		272 (53.0%)	270 (52.6%)	
N1	330 (34.6%)	187 (36.5%)		187 (36.5%)	187 (36.5%)	
N2	63 (6.6%)	35 (6.8%)		35 (6.8%)	35 (6.8%)	
N3	11 (1.2%)	6 (1.2%)		4 (0.8%)	6 (1.2%)	
M-classification			0.64			1.00
MX	22 (2.3%)	16 (3.1%)		15 (2.9%)	16 (3.1%)	
M0	911 (95.6%)	487 (94.9%)		485 (94.5%)	487 (94.9%)	
M1	20 (2.1%)	10 (1.9%)		13 (2.5%)	10 (1.9%)	
Thoracic surgical approach			0.58			1.00
Right-sided thoracotomy	731 (76.7%)	398 (77.6%)		406 (79.1%)	398 (77.6%)	
Thoracoscopy	152 (15.9%)	69 (13.5%)		62 (12.1%)	69 (13.5%)	
Other	70 (7.4%)	46 (9.0%)		45 (8.5%)	46 (9.0%)	
Neoadjuvant treatment			0.050			1.00
Yes	636 (66.7%)	316 (61.6%)		328 (63.9%)	316 (61.6%)	
No	313 (32.8%)	195 (38.0%)		184 (35.9%)	195 (38.0%)	
Missing	4 (0.4%)	2 (0.4%)		1 (0.2%)	2 (0.4%)	
Outcome—90 day Mortality	48 (5.0%)	34 (6.6%)	0.25	31 (6.0%)	34 (6.6%)	0.798

*BB* β-blockade, *CCI* Charlson’s Comorbidity Index, ASA American society of anesthesiologists

The β-blocker exposed group had a higher proportion of patients with a CCI ≥ 7 points (16.4% vs. 5.1%, p < 0.001) (Table 2). Specifically, β-blocker exposed patients had a significantly higher prevalence of preoperative cardiovascular diseases (arrythmia, myocardial infarction, congestive heart failure and hypertension, p < 0.001) (Table 3). No difference in crude 90-day postoperative mortality was detected between the groups (5.5% vs. 6.6%, p = 0.25).

**Table 3:** Preoperative comorbidities prior to and following propensity score matching

Preoperative Co-morbidity	Before matching			After matching		
	BB^(−)^	BB^(+)^	*p*	BB^(−)^	BB^(+)^	*p*
	(*n* = 953)	(*n* = 513)		(*n* = 513)	(*n* = 513)	
Arrythmia	169 (17.7%)	186 (36.3%)	< 0.001	119 (23.2%)	186 (36.3%)	< 0.001
Myocardial Infarction	20 (2.1%)	97 (18.9%)	< 0.001	15 (2.9%)	97 (18.9%)	< 0.001
Congestive heart failure	38 (4.0%)	80 (15.6%)	< 0.001	30 (5.8%)	80 (15.6%)	< 0.001
Hypertension	266 (27.9%)	403 (78.6%)	< 0.001	178 (34.7%)	403 (78.6%)	< 0.001
Peripheral vascular disease	23 (2.4%)	23 (4.5%)	0.044	19 (3.7%)	23 (4.5%)	0.635
Cerebrovascular disease	39 (4.1%)	40 (7.8%)	0.004	31 (6.0%)	40 (7.8%)	0.313
Peptic ulcer	48 (5%)	44 (8.6%)	0.011	33 (6.4%)	44 (8.6%)	0.242
Diabetes Mellitus	68 (7.1%)	59 (11.5%)	0.006	48 (9.4%)	59 (11.5%)	0.300
COPD	83 (8.7%)	59 (11.5%)	0.10	64 (12.5%)	59 (11.5%)	0.693
Liver diesease	18 (1.9%)	15 (2.9%)	0.28	13 (2.5%)	15 (2.9%)	0.850
Solid tumor	938 (98.4%)	489 (95.3%)	< 0.001	506 (98.6%)	489 (95.3%)	0.004
Leukemia	5 (0.5%)	1 (0.2%)	0.67	4 (0.8%)	1 (0.2%)	0.371
Lymphoma	6 (0.6%)	3 (0.6%)	1.00	5 (1.0%)	3 (0.6%)	0.724
Dementia	4 (0.4%)	2 (0.4%)	1.00	3 (0.6%)	2 (0.4%)	1.00

*COPD* chronic obstructive pulmonary disease

After propensity score matching, a total of 513 matched pairs were available for comparison. After matching there were no statistically differences between the groups for the matched covariates (Table 2). However, the β-blocker exposed group exhibited a higher incidence of cardiovascular diseases (Table 3). There was no statistically significant difference detected in 90-day postoperative mortality between the matched groups (6.0% vs. 6.6%, p = 0.798) (Table 2). This was validated by the conditional Poisson regression analysis which did not find a statistically significant benefit to β-blocker therapy in regard to 90-day postoperative mortality (incidence rate ratio: 1.17, 95% confidence interval, 0.55–2.46, p = 0.684).

## Discussion

To the best knowledge of the authors, this retrospective cohort study is the first study based on a nationwide patient inclusion for the assessment of the possible short-term survival benefit of β-blocker therapy following esophageal cancer surgery. The outlined results demonstrate no short-term survival benefit coupled to regular β-blocker therapy after esophageal resection surgery.

The stress response occurring at time of tissue injury is an essential physiological function and has been a subject of interest for scientists since the 1940s [4, 14, 15]. It is characterized by the release of catecholamines and their receptors and it is closely regulated by the sympathetic nervous system. A significant amount of stress on the body is induced by surgical trauma causing an activation cascade of interleukins that mediate inflammation and modulate immunity. Interleukins provide a positive feedback mechanism that activates a cascade of pro-inflammatory factors [16, 17]. Surgical trauma also activates a major catecholamine release, causing metabolic and hormonal changes in a multimodal systemic reaction which leads to insulin resistance, increased cytokine production, acute phase reactions, and lymphocytic proliferation with subsequent immunomodulatory changes [4]. This chain of events has a negative effect on the cardiovascular system in particular. Lindenauer et al. have shown an association between perioperative β-blocker treatment and the risk of death, as well as that this effect varies with patients’ cardiac risk [6]. Their retrospective cohort study, based on 782,969 patients from 329 hospitals, concluded that the use of β-blockers in cardiovascular high-risk surgical patients reduces in-hospital deaths. Likewise, in a study of 8351 patients, Devereaux et al. demonstrated that myocardial infarction after non-cardiac surgery occurs in five percent of patients, with nearly 75% of MIs taking place within the first 48 h [18]. Cardiac optimization of surgical patients is therefore of high relevance in attempts to reduce postoperative mortality.

Furthermore several retrospective cohort and prospective randomized studies suggest an association between β-blocker use and decreased postoperative morbidity and mortality following non-cardiac surgery [7, 8, 19, 20]. Mangano et al. conducted a randomized double-blinded placebo controlled study where they showed a survival benefit up to two years after surgery for patients who had a high cardiac risk and were maintained on peri- and postoperative β-blocker therapy compared to patients whose β-blocker was discontinued [21]. Likewise, patients on preoperative β-blocker therapy who undergo surgery for colorectal cancer show a decrease in postoperative complications and increased survival rates up to one year after surgery [5, 22]. However, there is paucity in the scientific evidence for the use of preoperative β-blockers in esophageal tumor surgery.

Several studies have shown an association between thoracic epidural anesthesia (TEA) after esophageal surgery and decreased postoperative complications and mortality [9, 23, 24]. Whooley et al. noticed a reduction in postoperative complications and in-hospital deaths correlating it to increased postoperative epidural analgesia and bronchoscopy [23]. These findings have been reinforced by other investigations where decreased cardiopulmonary complications and mortality in patients receiving adequate postoperative analgesia after transthoracic esophagectomy has been investigated [24]. It has been postulated that these positive findings are, to a large extent, due to the downregulation of the stress reaction caused by the surgical trauma which is also the proposed mechanism of action of β-blockers. In addition to decreasing stress-induced pain which facilitates early mobility after surgery, TEA is also postulated to have several overlapping beneficial effects with β-blockers such as decreasing myocardial oxygen consumption and the overall stress reaction [9]. Overwhelming majority of patients undergoing esophageal cancer surgery receive a TEA in Sweden.

The current study hypothesized that β-blockade in the context of surgery for esophageal cancer would have positive effects on short-term postoperative mortality. Despite the fact that the β-blocker exposed group in the current study was older, less fit for surgery and of higher comorbidity burden compared to the β-blocker unexposed group, no increase in crude short-term mortality was observed. Not surprisingly, the β-blocker exposed group had a significantly higher rate of cardiovascular diseases, which would increase their risk of adverse postoperative cardiac outcomes, and for that reason the continuation of β-blockers should be undertaken after esophageal resection surgery.

There is growing heterogeneity in publications outlining the impact of preoperative β-blocker therapy and clinical outcomes following non-cardiac surgery, especially when used in combination with TEA. Preoperative β-blockade in the context of laparoscopic gastric-by-pass surgery have not shown any difference in postoperative morbidity or mortality [25]. The presence of adverse effects such as stroke or death following β-blocker treatment in surgical patients have been recorded [26]. In contrast to the POISE (Peri Operative Ischemic Evaluation) trial in 2008 [26], several studies have shown an association between decreased mortality and β-blocker therapy for patients undergoing non-cardiac surgery [4–7, 19, 27]. Unlike the POISE trial, it can be assumed that the β-blocker therapy received by each patient in the current study was in a dose applied for each specific individual in contrast to the POISE trial where β-blocker “naïve” patients received a high dose of long-acting metoprolol despite major differences in co-morbidities, reason for surgery and the surgical procedure performed [26]. In contrast to the POISE trial, the strength of the current study is that it compares the effect of β-blocker therapy in a more homogenous surgical study cohort, i.e. including patients with the same diagnosis and treatment, who are subjected to the same degree of surgical trauma.

The authors recognize that there are limitations to this study due to its nature as a retrospective cohort investigation and the inability to ensure continuation of beta-blocker directly in the postoperative period and the synergic effect of β-blocker and thoracic epidural anesthesia. Another limitation is that no adjustments were made for the potential difference in the abdominal approach of the surgical procedures since this aspect was not registered in the majority of cases. In elderly patients, it has historically been more common for surgeons to choose an open technique for the abdominal part of the resection. However, in recent years there has been a shift towards laparoscopic surgery which is believed to decrease the surgical insult and thereby reduce the physiological stress induced by the open approach [28]. The majority of patients included in this study underwent a transthoracic approach irrespective of whether there was any concomitant exposure to β-blocker therapy or not, without any difference in the surgical approach between groups. Also, during the last few years a move towards centralization of cancer surgery has swept throughout Sweden. It is, however, not specified from the registry whether a patient was operated on in a low- or high-volume center at a specific time. The use of Enhanced Recovery After Surgery (ERAS) protocols is more common nowadays than it was 10 years ago. This is a weakness in the methodology since ERAS has a clear association with improved clinical outcomes [29, 30]. Nonetheless, these changes will be likely to result in equal effects on patients exposed and unexposed to β-blockers.

Furthermore, it was not possible to obtain data on the length of stay in ICU nor any sporadic doses of β-blocker or counteracting vasopressor agents given during the perioperative period. The study is also unable to account for optimal dose and timing of drug administration to conclude whether the outlined results are a cause of preoperative β-blockade only or the continuation of the drug postoperatively. In the current study cohort, the majority of patients are ASA class I-II, which puts them in a low cardiovascular risk. The beneficial effects of beta-blockers are shown to be more prominent in patients with moderate or high cardiovascular risk. However, the distribution of ASA I and II is similar in both the beta-blocker exposed and unexposed groups. In addition, the use of a nationwide registry results in a large cohort with wide heterogeneity. This has, to some extent, been compensated for by propensity score matching. There will, however, undoubtably be some residual bias despite this statistical methodology. Propensity score matching, which has been developed to be a surrogate for retrospective randomization, does only take into account variables known to be associated with outcomes, which necessitates a prospective randomized clinical trial. A randomized controlled trial is also required to ensure a fully effective control group who differ only in beta-blocker exposure.

## Conclusion

In conclusion, this study supports the continued use of preoperative β-blocker therapy in patients undergoing esophageal cancer resection surgery who have a pre-existing need for β-blocker therapy without increasing the risk of post-operative mortality following surgery. The potential therapeutic implication of its use in β-blocker naïve patients and its synergic effect with TEA cannot be commented on in the current study and need further investigation.

## Data Availability

The data used for this manuscript can be provided for reasonable request to the editorial office after IRB approval.

## Abbreviations

ACC/AHA: American College of Cardiology and American Heart Association
ASA: American Society of Anesthesiologist classification
ATC: Anatomical Therapeutic Chemical Classifications
BB: β-Blocker
CCI: Charlson Comorbidity Index
CI: Confidence interval
COPD: Chronic obstructive pulmonary disease
ICD-codes: International Statistical Classification of Diseases
IRR: Incidence rate ratio
NREV: Swedish National Register for Esophagus and Gastric Cancers
SD: Standard deviation
TEA: Epidural thoracic analgesia
TNM: Tumor, nodes, metastasis classification
